# Screening for depression in children and adolescents: a protocol for a systematic review update

**DOI:** 10.1186/s13643-020-01568-3

**Published:** 2021-01-12

**Authors:** Andrew Beck, John C. LeBlanc, Kate Morissette, Candyce Hamel, Becky Skidmore, Heather Colquhoun, Eddy Lang, Ainsley Moore, John J. Riva, Brett D. Thombs, Scott Patten, Heather Bragg, Ian Colman, Gary S. Goldfield, Stuart Gordon Nicholls, Kathleen Pajer, Beth K. Potter, Robert Meeder, Priya Vasa, Brian Hutton, Beverley J. Shea, Eva Graham, Julian Little, David Moher, Adrienne Stevens

**Affiliations:** 1 Knowledge Synthesis Group, Clinical Epidemiology Program, Ottawa Hospital Research Institute, Centre for Practice-Changing Research, 501 Smyth Road, Box 201, Ottawa, ON K1H 8L6 Canada; 2 Department of Pediatrics, Dalhousie University, Halifax, NS Canada; 3 Public Health Agency of Canada, Ottawa, ON Canada; 4 Department of Occupational Science and Occupational Therapy, University of Toronto, Toronto, ON Canada; 5 University of Calgary Cumming School of Medicine, Calgary, AB Canada; 6 Alberta Health Services, Calgary, AB Canada; 7 Department of Health Research Methods, Evidence and Impact, McMaster University, Hamilton, ON Canada; 8 Department of Family Medicine, McMaster University, David Braley Health Sciences Centre, Hamilton, ON Canada; 9 Lady Davis Institute of the Jewish General Hospital, Montreal, QC Canada; 10 Faculty of Medicine, McGill University, Montreal, QC Canada; 11 Department of Community Health Services and Department of Psychiatry, University of Calgary, Calgary, AB Canada; 12 Children’s Hospital of Eastern Ontario, Ottawa, ON Canada; 13 School of Epidemiology and Public Health, Faculty of Medicine, University of Ottawa, Ottawa, ON Canada; 14 Children’s Hospital of Eastern Ontario Research Institute, Ottawa, ON Canada; 15 Ottawa Hospital Research Institute, Ottawa, ON Canada; 16 Department of Psychiatry, Children’s Hospital of Eastern Ontario, Ottawa Faculty of Medicine, Ottawa, ON Canada; 17 Waypoint Centre For Mental Health Care, Penetanguishene, ON Canada; 18 Department of Family and Community Medicine, St. Michael’s Hospital, University of Toronto, Toronto, ON Canada

**Keywords:** Depression, screening, systematic review, child, children, adolescent, youth

## Abstract

**Background:**

Major depressive disorder is common, debilitating, and affects feelings, thoughts, mood, and behaviors. Childhood and adolescence are critical periods for the development of depression and adolescence is marked by an increased incidence of mental health disorders. This protocol outlines the planned scope and methods for a systematic review update that will evaluate the benefits and harms of screening for depression in children and adolescents.

**Methods:**

This review will update a previously published systematic review by Roseman and colleagues. Eligible studies are randomized controlled trials (RCTs) assessing formal screening in primary care to identify children or adolescents not already self-reporting symptoms of, diagnosed with, or treated for depression. If no or only a single RCT is available, we will consider controlled studies without random assignment. Studies of participants with characteristics associated with an elevated risk of depression will be analyzed separately. Outcomes of interest are symptoms of depression, classification of major depressive disorder based on a validated diagnostic interview, suicidality, health-related quality of life, social function, impact on lifestyle behavior (e.g., substance use, school performance, lost time at work, or school), false-positive results, overdiagnosis, overtreatment, labeling, and other harms such as those arising from treatment. We will search MEDLINE, Embase, PsycINFO, CINAHL, the Cochrane Library, and grey literature sources. Two reviewers will independently screen the titles and abstracts using the liberal accelerated method. Full-text screening will be performed independently by two reviewers using pre-specified eligibility criteria. Data extraction and risk of bias assessments will be performed independently by two reviewers. Pre-planned analyses, including subgroup and sensitivity analyses, are detailed within this protocol. Two independent reviewers will assess and finalize through consensus the certainty of evidence using the Grading of Recommendations Assessment, Development and Evaluation (GRADE) approach, and prepare GRADE evidence profiles and summary of findings tables for each outcome of interest.

**Discussion:**

The systematic review will provide a current state of the evidence of benefits and harms of depression screening in children and adolescents. These findings will be used by the Canadian Task Force on Preventive Health Care to inform the development of recommendations on depression screening.

**Systematic review registration:**

PROSPERO CRD42020150373

**Supplementary Information:**

The online version contains supplementary material available at 10.1186/s13643-020-01568-3.

## Background

Major depressive disorder (MDD) is a common, debilitating mood disorder characterized by negative feelings, thoughts, and behaviors that causes significant impairment in social, occupational and educational functioning, and quality of life, and is related to an increased risk of suicide and death [1, 2]. During the transition from childhood to adolescence, there is an increase in prevalence of some psychiatric disorders including depression [3]. This transition involves physical, psychological, and emotional changes typical of this developmental period, which may increase an individual’s sensitivity and reactivity to stress exposure [4, 5]. As with the adult population, diagnoses of depressive episodes in children and adolescents are established by one of the two commonly used diagnostic classification systems for psychiatric diagnoses, the Diagnostic and Statistical Manual of Mental Disorders, Fifth Edition (DSM-5) [6], and International Classification of Diseases, 10^th^ Revision (ICD-10) [7]. Each diagnostic system provides a minimum number of criteria that must be met over a 2-week period for an episode to be considered a depressive episode. Also, the DSM-5 includes further criteria to specifically define MDD for children and youth [6]. Symptoms of irritability can be considered in place of depressed mood, and a failure to meet expected weight gain can be considered instead of weight loss (see Additional file 1).

### Prevalence and burden

From the 2014 Ontario Child Health Study, the 6-month prevalence of possible major depressive episodes (MDE) was 1.1% for children (4- to 11-year-old) and 5.2% or 7.5% for adolescents (12- to 17-years-old) based on parent or adolescent report, respectively [8]. In pooled estimates from the Canadian Community Health Survey, a series of cross-sectional surveys from 2000 to 2014, 5.5% of 12- to 19-year-olds reported experiencing MDE-like episodes in the past year, with little change in prevalence from 2000 to 2014 [9]. Rates were higher among females and among those aged 15 to 19 years (10.1% for females; 4.1% for males) compared to those aged 12 to 14 years (4.1% for females; 0.6% for males) [9], with similar findings supported by other literature [10–12].

The burden of depression is high among children and adolescents. Unipolar depressive disorders (i.e., major depressive episode, dysthymia) are a leading cause of years lost to disability among both the 10- to 14-year-old and 15- to 19-year-old age groups [13, 14]. Poor long-term social outcomes are also a consequence of depression in adolescence. Those with depression are at an increased risk of leaving secondary school, unemployment, early pregnancy, and parenthood [15]. As well, they have a lower likelihood of entering post-secondary education [15]. Depression with onset in childhood and adolescence can continue into adulthood, posing a burden on individuals, families, and communities [15–18]. A recent systematic review found that adolescents who suffer from depression have around 2.5 (2.78 [95% CI 1.97, 3.93]) times the odds of developing depression in adulthood compared with adolescents without depression [16]. Additionally, those who suffer from depression in adolescence are at an increased risk for suicidal ideation, attempts, and completion in adulthood [19–21].

### Risk factors

There are several risk factors associated with depression in children and adolescents. As shown above, females are at a higher risk, particularly in adolescence, with the difference between sexes becoming smaller later in adulthood [12, 22]. A family history of depression and exposure to adverse events such as illness or death of a family member, physical or sexual abuse, are also strong risk factors [23, 24]. Parental behaviors associated with an increased risk include aversive behaviors toward the child or adolescent (e.g., criticism, punishment, and conflict), lack of autonomy given to the child or adolescent, lack of warmth, inconsistent parental discipline, and parental over-involvement [25]. Other influential factors include aspects related to the school environment such as bullying, low connectedness with peers and teachers [26, 27], poor academic achievement [28], learning disabilities (e.g., attention deficit hyperactivity disorder, dyslexia) [29–31], and community environment factors such as safety, ethnicity, and prevalence of discrimination [32]. Additional risk factors include substance abuse (e.g., alcohol, tobacco, cannabis, other illicit drugs), poor sleep, screen time, unhealthy diet, and weight problems [33–35].

### Screening of MDD in children and adolescents

A screening program for depression must identify symptomatic disease that would not otherwise be reported (e.g., by spontaneous patient self-report, parent/caregiver report, or clinical inquiry). If effective, screening for depression would be expected to lead to interventions that improve future health outcomes in those who otherwise would not have been identified [36]. However, as noted by Cosgrove et al. [37], without evidence on the benefits and harms of screening, there are several assumptions that are questionable in the case of depression screening. First, unlike other disorders, depression does not have a detectable asymptomatic early stage. It manifests as one or more discrete episodes and many patients remit after an initial episode. Screening tools rely on identifying symptoms of depression itself and therefore can only be effective at early detection if the use of the tool prompts consideration of whether or not symptoms of depression are present. Second, there is currently little evidence that adding screening questionnaires to primary care reduces depressive symptoms. Third, optimal treatment for screen-detected depression is not clear. Most people identified as depressed via screening will have mild symptoms that may resolve without intervention. Many are treated with antidepressant medications. However, the majority of antidepressant medications have not been shown to be as effective in adolescents as in adults, may increase risk for suicidality, and may be even less likely to be effective for the mild cases likely overrepresented in patients identified through screening questionnaires [38, 39]. Psychological therapies are a reasonable option to medications with some positive effects [40, 41].

In considering the potential benefits and harms of screening, the proper design of trials is paramount [42, 43]. Experts have criticized previous systematic reviews on depression screening that have not explicitly defined the characteristics of screening trials to isolate the effect of screening from, for example, diagnostic suspicion by patients or clinicians [44, 45]. For example, a screening trial should separate the effect of screening from the effect of providing additional treatment resources not otherwise available. Since screening programs should only identify previously unrecognized cases, a screening trial should also exclude patients already diagnosed or under care for depression [42]. Accordingly, we have specified criteria that would need to be met to reduce selection and confounding bias in the inclusion of evidence. These are detailed in the “Methods” section.

### Previous guideline recommendations using systematic reviews from the Canadian Task Force on Preventive Health Care and other guideline developers

In 2005, the Canadian Task Force on Preventive Health Care (“Task Force”) published recommendations on screening for depression in the adult population as well as children and adolescents. The Task Force guideline concluded that there was insufficient evidence to recommend either for or against depression screening in children and adolescents [46]. In 2016, the U.S. Preventive Services Task Force (USPSTF) updated their 2009 guideline and systematic review and recommended routinely screening for depression in adolescents (age 12 to 18 years) in primary care settings “with adequate systems in place to ensure accurate diagnosis, effective treatment, and appropriate follow-up,” but not in children (aged ≤ 11 years) due to insufficient evidence [47, 48]. The USPSTF recommendation for screening relied on indirect and linked evidence that screening instruments for depression can accurately identify MDD in adolescents and that treatment of MDD detected through screening in adolescents is associated with moderate benefit. However, this was not based on screening trials, as no trials were identified that directly assessed the effects of screening compared with no screening [48]. The recommendation was based on eight “fair- “or “good-quality” placebo-controlled intervention trials examining the effectiveness of antidepressants, psychotherapy, and collaborative care interventions (e.g., the Massachusetts Child Psychiatry Access Project, an inter-professional collaboration among primary care providers and mental health specialists) for children and adolescents with MDD. They found that one trial had shown effectiveness with fluoxetine as well as combined fluoxetine and cognitive behavioral therapy. Furthermore, one of the two escitalopram trials and the one collaborative care trial had demonstrated benefits. The USPSTF considered the evidence on screening test accuracy and treatment of MDD adequate and the harms of pharmacotherapy treatment to be minimal if patients were closely monitored. Similar to the USPSTF, the Guidelines for Adolescent Depression in Primary Care (GLAD-PC) Steering Group updated their 2007 systematic literature review and guideline [49] and found no evidence comparing screening to no screening or usual care in primary care settings [50]. They relied upon indirect evidence surrounding the validity and feasibility of screening tools and the feasibility and effectiveness of treatment for individuals with depression. GLAD-PC recommended annual screening for depression in adolescents (ages 12 and older) based on psychometric data on depression screening tools as well as research on screening issues (e.g., whether screening is occurring and whether screening impacts follow-up procedures or treatment to the specifics of screening). They considered that early identification and treatment of adolescent depression is important because of the high prevalence of depression and its persistence, its potential occurrence during a crucial brain development period, and the potential consequences with transition into adulthood. Among those with depression risk factors ages 10 to 21 years, GLAD-PC recommended not only annual screening but also more frequent targeted screening (i.e., systematically monitoring individuals over time for the development of a depressive disorder) during other health care visits. Their justification for this recommendation was that these individuals are likely to experience future depressive episodes and those who are not diagnosed with depression but report high scores on the screening tools may be at risk for depression within 6 months [50].

### Rationale

There are inconsistencies among the current screening guidelines reviewed above, notably their reliance on indirect evidence and the failure to include RCTs where the potential impacts of screening and treatment are clearly separated. A recent review by Roseman and colleagues [51] was selected by the Task Force working group as an appropriate review to update but with modifications, such as also addressing patients that may have an elevated risk of depression, including other outcomes of relevance to decision making, other study design filters, and expanding the search approach. This review update will provide a current assessment of the evidence for the Task Force guideline recommendations.

## Review objective and key questions

The Task Force is undertaking a systematic evaluation of the literature to inform its upcoming guideline recommendation on depression screening in children (6 to 11 years old) and adolescents (12 to 17 years old) in primary care. A depression working group of task force members (i.e., guideline panel) and external clinical experts was formed. The development of the topic, refinement of the key questions, and the structured Population, Intervention, Comparator, Outcome, Study Design (PICOS) eligibility framework involved discussions among the working group and members of the Public Health Agency of Canada (PHAC) and the Ottawa Evidence Review and Synthesis Centre (ERSC). For more information on the process of topic development and selection of working group members and clinical experts, please refer to the Task Force Procedure Manual (https://canadiantaskforce.ca/methods/).

The purpose of this systematic review is to examine the evidence on screening for depression in children and adolescents within primary care, and the findings will be used by the Task Force to inform the development of guideline recommendations. This protocol outlines the methodological process for performing a review update of Roseman et al. (2017) [51].

Our systematic review update will be guided by the following key questions (KQ):

KQ1: What are the benefits and harms of screening for depression in children (6 to 11 years old) and adolescents (12 to 17 years old) in primary care or other non-mental health clinic settings?

KQ1a: What are the benefits and harms of screening for depression in children (6 to 11 years old) and adolescents (12 to 17 years old) in primary care or other non-mental health clinic settings for patients targeted because they have characteristics that may suggest elevated risk of depression?

**Fig. 1:**
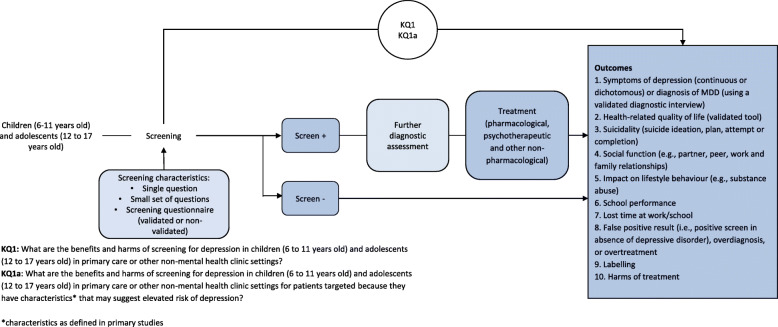
Analytic framework

Figure 1 presents an analytic framework of screening for depression in children and adolescents in primary care and the relevant health outcomes.

After reviewing the evidence pertinent to KQ1 and KQ1a, the working group may consider a separate systematic review on the patient values and preferences to inform their guideline beyond what is known from their usual patient engagement activities described in the following sections. The decision to undertake a systematic review on values and preferences will be directed by the Grading of Recommendations Assessment, Development, and Evaluation (GRADE) methods and will weigh all a priori identified critical outcomes for both benefits and harms of screening. If the decision is made to pursue a values and preferences review on KQ2 and KQ2a, a separate protocol will be developed at that time that would include topic refinement with the working group. The potential key questions are:

KQ2. In children and adolescents without characteristics that may suggest elevated risk of depression, how do they (or their parents/guardians) value outcomes that may occur from screening for depression, and how do these values influence decisions about being screened?

KQ2a. In children and adolescents with characteristics that may suggest elevated risk of depression, how do they (or their parents/guardians) value outcomes that may occur from screening for depression in children and adolescents, and how do these values influence decisions about being screened?

## Methods

The review will be completed by the ERSC at the Ottawa Hospital Research Institute. Internal methodological experts, clinical experts, and a patient advisor will be consulted at key points during the conduct of the review. The systematic review will be developed, conducted, and prepared according to the Task Force Procedure Manual [52].

### Protocol and registration

This protocol was developed by the ERSC and reviewed by their clinical experts and patient representative, the Depression working group members and their external clinical experts, the PHAC, Task Force members, and stakeholder organizations.

The protocol was prepared following the Preferred Reporting Items for Systematic Reviews and Meta-Analyses Protocols (PRISMA-P checklist) [53] (Additional file 2). The project is registered with the International Prospective Registry of Systematic Reviews (PROSPERO) database (CRD42020150373). The final review will be reported using the Preferred Reporting Items for Systematic Reviews and Meta-Analyses (PRISMA) checklist and the A Measurement Tool to Assess Systematic Reviews (AMSTAR 2) tool will be used for additional quality control [54, 55]. We will document any amendments to this study protocol in PROSPERO and present them in the final manuscript.

### Eligibility criteria

Studies for the review will be selected for inclusion according to the eligibility criteria presented in Table 1. Randomized controlled trials (RCTs) examining depression screening tools among patients who are up to 17 years of age will be eligible. If no or only a single RCT is available, controlled studies without random assignment will be sought. Studies that include other ages (e.g., 6 to 20 years of age), but present results separately for those up to 17 will be included and those that do not present results separately for the relevant ages will be excluded. Children will be defined as 6 to 11 years of age and adolescents will be defined as 12 to 17 years of age. For KQ1a, the target population will include patients who are selected for screening because they have characteristics that suggest elevated risk of depression. In all cases, we will include studies where caregivers respond to screening tool questions on behalf of children.

**Table 1:** Inclusion and exclusion criteria for key question 1 and 1a

	Inclusion	Exclusion
Population	KQ1: Patients who are up to and including 17 years of age. KQ1a: Patients who are up to and including 17 years of age selected for screening because they have characteristics that may suggest elevated risk of depression. For both key questions, caregivers may respond to screening questions on behalf of children. Onset of adolescence will be considered as being age 12. *Characteristics as defined in primary studies*	• > 20% of the study sample are adults (18 years and older), have a recent history of depression, have a current diagnosis, or are receiving treatment for depression or other mental disorders (unless results are provided separately from the sample of interest) • Members of the study sample are seeking services due to symptoms of mental disorders • Members of the study sample are receiving assessment or care in psychiatric or mental health settings • Members of the study sample are currently pregnant or have given birth in the past year
Intervention	Screening tools that use a single question, a small set of questions, or a screening questionnaire (validated or non-validated) with a pre-defined cut-off score to identify patients who may have depression, but who have not previously reported their symptoms to healthcare providers or who have otherwise not been identified as possibly depressed by healthcare providers. *Patients and/or their guardians have the ability to answer screening tool questions*	Screening tools that, in addition to screening, include depression care referral or treatment options not available to patients identified as depressed in the non-screening trial arm
Comparator	No depression screening. • Patients in comparator arms may be administered depression symptom questionnaires for the purpose of baseline or outcome assessments as long as scores are not provided to the patients or healthcare providers prior to start of intervention.	
Outcomes	Critical 1. Symptoms of depression (measured continuously or dichotomously) or diagnosis of MDD (using a validated diagnostic interview) 2. Health-related quality of life (validated tool) 3. Suicidality (suicide ideation, plan, attempt or completion) 4. Social function (e.g., partner, peer, work and family relationships) 5. Impact on lifestyle behavior (e.g., substance abuse) Important 6. School performance 7. Lost time at work/school 8. False-positive result (i.e., positive screen in absence of depressive disorder), overdiagnosis, or overtreatment 9. Labeling 10. Harms of treatment	
Setting	Primary care or non-mental health clinic settings, such as medical specialist clinics, schools or recreational/community settings, and online settings (e.g., online depression screening), where screening is administered by a health practitioner	Studies conducted in mental health, or psychiatric settings. Studies in non-mental health clinic settings where screening is administered by a non-health practitioner.
Study design	Randomized controlled trials (RCTs), including cluster-randomized trials^a^ If no or only a single RCT is available, then controlled studies without random assignment^b^	RCTs where patient eligibility is determined, and patients are enrolled after randomization. Interrupted times series, single cohort studies, case-control studies, cross-sectional studies, case series, case reports, and other publication types (editorials, commentaries, notes, letter, opinions).
Publication language	English or French	Languages other than English and French.
Dates of publication	January 2017 to present (RCTs) January 2015 to present (non-randomized controlled studies)	

^a^Eligible RCTs will need to meet the following criteria: wherein patient eligibility is determined and then patients are enrolled prior to randomization (i.e., to screening or to no screening); similar resources for depression management and treatment must be available both to patients in the screening arm of the trial and to patients in the non-screening arm of the trial who are identified as depressed via other methods (e.g., unaided clinician diagnosis, patient report) [43]

^b^Eligible nonrandomized controlled studies will need to meet the following criteria: Similar resources for depression management and treatment must be available both to patients in the screening arm of the trial and to patients in the non-screening arm of the trial who are identified as depressed via other methods (e.g., unaided clinician diagnosis, patient report) [43]

The depression screening tool can be a single question, a small set of questions, or a screening questionnaire (validated or not) with a pre-defined cut-off score to identify patients who may have depression but who have not reported their symptoms to healthcare providers or who have otherwise not been identified as possibly depressed by healthcare providers. The eligible comparator will be no depression screening. Both the screening and no screening arms will offer the same interventions for patients identified with depression symptoms. We will consider “no screening” to reflect normal clinical care that requires ongoing active observation and conversation with patients around symptoms suggestive of depression but no use of a formalized screening instrument. Depression screening should involve administering a depression symptom questionnaire to all patients and using a pre-identified cut-off threshold to classify patients as having positive or negative screening results. Accordingly, the use of a screening tool without the consideration of a pre-defined cut-off threshold would not be considered screening. Those with positive screening results can then be evaluated to determine if they have depression and, if appropriate, be offered treatment.

Studies in primary care or non-mental health clinic settings (e.g., medical specialist clinics, schools, or recreational/community settings) and online settings where screening is administered by a health practitioner will be eligible. Since this is a review update from Roseman et al. [51], we will continue to limit to RCTs and include reports from January 2017 onwards. If non-randomized controlled studies are sought, we will search from January 2015 onwards and replace the RCT study filter with a non-randomized controlled study design filter. The search date will be based on the latest search from the 2016 USPSTF review (February 2015), which did not find any non-randomized controlled studies assessing the effects of screening [48]. Their search would be difficult to replicate as it used one search for multiple key questions that were not relevant for this project. However, the USPSTF had similar eligibility criteria to ours for their key question on the effectiveness of screening and we will consider their list of excluded studies to ensure all studies of interest are captured. Reports will be restricted to those written in English and French. Previous literature suggests including other languages when reviewing topics such as complementary and alternative medicines interventions [56]. We will use the following inclusion criteria from a recent depression screening systematic review to determine eligible RCTs and controlled studies without random assignment, where possible [43, 57]: (1) the patient population is clearly defined and randomized prior to administering the screening test (only applies to RCTs); (2) patients known to have a diagnosis of depression, or who are being treated for depression are excluded (screening is intended to identify undetected cases, and those who are known to have depression should not be screened in clinical practice); and (3) similar resources for depression management and treatment must be available both to patients in the screening arm of the trial and to patients in the non-screening arm of the trial who are identified as depressed via other methods (e.g., unaided clinician diagnosis, patient report).

### Outcome rating

Members of the Depression working group developed the list of preliminary outcomes of interest, presented in Table 1. Through consensus, those outcomes were rated by the six working group members according to GRADE methodology as critical (rated 7 to 9 out of 9), important (rated 4 to 6 out of 9), or of limited importance (rated 1 to 3 out of 9) for making guideline recommendations [58]; only critical and important outcomes will be included. These outcomes will also be rated, according to GRADE methodology, by relevant groups of patients who are unaware of the working group ratings through the Task Force patient engagement activities conducted by the Knowledge Translation Program at St. Michael’s Hospital in Toronto, Ontario. The list of outcomes will be finalized before final study selection and data extraction, and the PROSPERO record will be updated, as required. For more details on the outcome rating process, please see https://canadiantaskforce.ca/methods/ for the working group outcome rating and https://canadiantaskforce.ca/methods/patient-preferences-protocol/ for rating through patient engagement.

Outcomes will not be used in the selection of studies. Based on ratings to date by the six working group members, outcomes of interest deemed of critical importance for guideline development and decision-making are the symptoms of depression or diagnosis of MDD (average rating of 8.7), suicidality (average rating of 7.5), health-related quality of life (average rating of 6.8), social function (average rating of 6.5), and impacts on lifestyle behavior such as substance use (average rating of 6.5). Outcomes rated as important are school performance (average rating of 6.2), harms of treatment (average rating of 6.2), lost time at work or school (average rating of 5.5), false-positive result, overdiagnosis, or overtreatment (average rating 5.5), and labeling (average rating of 4.7). No outcomes were rated as limited importance. The outcomes are listed in Table 1.

### Information sources and search strategy

The search strategies were developed and tested through an iterative process by an experienced medical information specialist in consultation with the review team. Using the OVID platform, we will search Ovid MEDLINE®, including Epub Ahead of Print and In-Process & Other Non-Indexed Citations, Embase Classic+Embase, and PsycINFO. We will also search the Cumulative Index to Nursing and Allied Health Literature (CINAHL) (Ebsco) and CENTRAL (Wiley). The searches will utilize a combination of controlled vocabulary (e.g., “Depressive Disorder,” “Mass Screening,” “Adolescent”) and keywords (e.g., MDD, screen, pediatric). We will apply an amended version of the 2008 Cochrane Highly Sensitive Search Strategy, sensitivity- and precision-maximizing version, to identify randomized controlled trials, and an additional filter to identify non-randomized controlled studies. Vocabulary and syntax will be adjusted across the databases. There will be no language restrictions, but results will be limited to the period of 2017 to the present for RCTs and 2015 for non-randomized controlled studies. Animal-only and opinion pieces will be removed from the results.

The draft search strategy for the MEDLINE database was peer-reviewed by another experienced librarian using the Peer Review of Electronic Search Strategies (PRESS) checklist [59], modified where necessary, and then translated to other databases. The draft strategies are available in Additional file 3.

We will search grey literature sources to identify studies not formally published in journals and any ongoing trials using the Canadian Agency for Drugs and Technologies in Health (CADTH) Grey Matters checklist [60]. The CADTH checklist includes public health websites, health technology assessment agencies, clinical practice guideline organizations, clinical trials registries, search engines, and additional databases. We will also search the websites of the following organizations: American Academy of Child and Adolescent Psychiatry, American Academy of Family Physicians, American Academy of Pediatrics, American College of Physicians, American Nurses Association, American Psychological Association, Anxiety and Depression Association of America, Canadian Academy of Child and Adolescent Psychiatry, Canadian Mental Health Association, Canadian Nurses Association, Canadian Paediatric Society, Canadian Psychiatric Association, Centre for Addiction and Mental Health, Child Mind Institute, and the College of Family Physicians of Canada. Searches for grey literature will be restricted to English and French language documents and limited to a 5-day maximum search period by one person. The reference list of included studies and secondary evidence reports (e.g., evidence-based clinical practice guidelines, systematic reviews, and meta-analyses) will also be reviewed. Using Robinson et al. (2014) as guidance [61], a review would need to meet the following criteria to be considered systematic: (1) At least one database was searched; (2) authors report selection criteria; (3) risk of bias (or intended analogous) of included studies is reported; and (4) authors report a list and synthesis of included studies.

### Study selection

Citations from bibliographic databases, grey literature, and supplemental source searching will be collated in Reference Manager [62]. Those citations will be de-duplicated and uploaded to an online systematic review management software package, DistillerSR [63]. A pilot test will be conducted by two reviewers for title and abstract (random sample of 50 citations) and full-text levels (random sample of 25 articles) to test the screening questions and to ensure consistent application of the selection criteria among reviewers. Adjustments to the forms will be made as needed.

Title and abstract screening will be performed by two reviewers using the liberal accelerated method [64]. This approach involves one reviewer screening citations and a second reviewer verifying the citations excluded by the first reviewer. References will be screened in random order, and screening will be done concurrently among reviewers to reduce the likelihood that a citation has been already considered by another reviewer. Conflict resolution will not be conducted at this stage.

At the full-text screening stage, two reviewers will independently and in duplicate review the full text of potentially relevant articles to determine eligibility. Conflicts will be resolved by consensus or by consulting with a senior team member. Reviewers will agree on the reasons for exclusion at the full-text stage. 

Where study eligibility is unclear, authors will be contacted for additional information twice by email over three weeks. Where input is needed for potentially eligible studies, we will consult with working group members and clinical experts who are blinded to study identification information and outcome data. Articles that are not available electronically will be ordered via interlibrary loan, allowing 30 days for receipt. Potentially relevant studies available only in abstract form will be excluded and the citation will be annotated in the list of excluded studies. To reduce potential bias, we will consult with working group members and clinical experts on any potentially missing studies once all outstanding queries related to synthesizing the evidence are addressed. Draft screening forms are available in Additional file 4.

### Data extraction

Before commencing data extraction, we will pilot the data extraction form in DistillerSR on a random sample of full-text articles (three to six articles, depending on the number of included studies) and modify the form as needed. Two reviewers will independently extract data, compare data sets, discuss, and resolve any conflicts through discussion. If consensus cannot be reached, we will consult with a senior team member. Study authors will be contacted by email twice over 3 weeks for further information as required. We will extract all formats of continuous outcome data whether reported as post-intervention or change from baseline. Draft items for data extraction are provided in Additional file 5, and clinical experts and the working group will be consulted on extraction variables.

### Risk of bias in individual studies

Concurrently with data extraction, we will follow Task Force methods and will appraise the risk of bias (ROB) using the Cochrane ROB tool for RCTs [65] and the Newcastle-Ottawa Scale (NOS) for non-randomized controlled studies [66]. Two reviewers will independently assess the ROB of all included studies, compare assessments, and resolve any assessments and their supports for judgments where disagreement has occurred. This will be done by consensus or by consulting with a senior team member when required. The Cochrane ROB tool consists of the following seven domains: (1) random sequence generation, (2) allocation concealment, (3) blinding of participants and personnel, (4) blinding of outcome assessment, (5) incomplete outcome data, (6) selective reporting, and (7) other sources of bias. As recommended by the Cochrane Handbook [67], certain domains are outcome-specific (e.g., blinding of outcome assessors) and will be assessed at the outcome level. The overall ROB will require the consideration of the relative importance of the seven domains accompanied by the known empirical evidence of bias, likely direction of bias, and likely magnitude of bias [65]. For outcome and analysis reporting bias, we will use the methods outlined in the Agency for Healthcare Research and Quality guidance [68]. For assessing cluster randomized trials, we plan to assess recruitment bias (when trial recruitment follows after clusters have been randomized and the knowledge of intervention and control clusters could affect the types of participants recruited) in the “other risks of bias” domain of the Cochrane ROB tool [69]. We will classify the overall ROB as low if all domains were assessed at low ROB, high if at least one domain was assessed at high ROB, or unclear if at least one domain was assessed at unclear ROB and no domain was at high risk.

The NOS for cohort studies consists of the following three domains: (1) selection of study groups, (2) comparability of study groups, and (3) outcome assessment. Each item within the selection (4 items) and outcome (3 items) domains can be awarded a maximum of one point, and two points for the single item in the comparability domain. A study can be awarded a maximum of nine points for the least risk of bias [66]. We will classify good quality studies as scoring at least 3 stars in the selection domain, at least 1 star in comparability, and at least 2 stars in the outcome domain. A fair quality study will require 2 stars in selection, at least 1 star in comparability, and at least 2 stars in the outcome domain. A poor-quality study will score 0 or 1 star(s) in selection, or 0 stars in comparability, or 0 or 1 star(s) in the outcome domain.

The individual study ROB and NOS assessments will inform the study limitations domain assessment across studies for each outcome using GRADE assessments, described in the following sections [70].

### Synthesis of included studies and interpretation

We will describe and present in summary tables the study characteristics, participant characteristics, intervention and comparator details, outcome results, and Cochrane ROB or NOS evaluations. We will convert data (e.g., recovery of intervention group standard deviations from standard errors or 95% confidence intervals) to facilitate consistent presentation and synthesis of the results across studies. We will calculate the appropriate statistics based on the type of outcome data. Relative and absolute effects with 95% confidence intervals will be calculated to facilitate the presentation of outcome data according to the GRADE guidance for developing evidence profile tables and summary of findings. Risk ratios and risk differences will be used to report effects for binary data. When presenting continuous data, GRADE guidance will be used [71]. Where needed, we will use a conservative value for a correlation coefficient of 0.25 to impute standard deviations for means used in change from baseline calculations, as performed in other reviews [72]. Studies with a cluster design (e.g., allocation by clinical setting) with data analyzed at the individual level (e.g., patients) will provide incorrect estimates of precision if correlation within clusters is not taken into account [73]. Where authors of cluster randomized trials have not adjusted for unit-of-analysis errors, we will attempt to correct these by using intra-class correlation coefficients (ICC) when available and by estimating ICCs when not [67, 74]. Where possible, the number needed to screen will be calculated.

#### Meta-analysis

Prior to performing a meta-analysis, we will assess the clinical (e.g., patient characteristics) and methodological (e.g., study design) heterogeneity of included studies. We will seek input from clinical experts and the guideline working group on decision-making.

The *I*^2^ statistic will be used to assess the statistical heterogeneity across included studies (low [0–25%], moderate [25–50%], substantial [50–75%], and considerable [> 75%]) [75–77]. We will use the Cochran’s *Q* test (threshold *p* value < 0.10) to interpret the strength of evidence for heterogeneity [78, 79]. If considerable heterogeneity is observed (defined as *I*^2^ of > 75%), we will present studies in a forest plot, but will not report a pooled estimate. We will make efforts to explain statistical heterogeneity via the performance of subgroup analyses, sensitivity analyses, and/or meta-regression analyses; the optimal approach will be determined once determining what information is available. Meta-regression will be based on random effects models to allow for residual unexplained heterogeneity. We will follow previously published guidance for meta-regression [75]. The meta-regression analyses will be conducted when 10 or more studies are available for a continuous study-level variable. For a categorical subgroup variable, each subgroup should have four or more studies. These numbers serve as the lower bounds for consideration of meta-regression for small studies [75]. We will use univariable meta-regression when an insufficient number of studies is available to conduct multivariable analyses.

When a meta-analysis is appropriate (e.g., studies are similar and most do not have high ROB), we will use a random effects model for effect measures. Risk ratios and risk differences will be used for pooling binary outcomes. Mean differences (outcomes measure in a similar manner or with the same scale) or standardized mean differences (where outcomes measured using different scales) will be used for continuous data. We will not combine RCTs and non-randomized controlled studies in a meta-analysis. If a meta-analysis is not appropriate, we will present the range of effects.

#### Sparse binary data and studies with zero events

When studies report endpoints with low event rates, the results will be summarized narratively. The risk difference summary measure will be used for the outcomes where at least one intervention group contains zero events (e.g., suicide completion). We will use the median baseline risk for the control group, where available, for risk difference calculations to enable reporting for GRADE tables. However, we may conduct a sensitivity analysis using different baseline risks if suitable.

#### Subgroup analysis

If there are sufficient data and adequate reporting, the following subgroup analyses are planned:

- Socioeconomic status of parent(s) (e.g., income, level of education; as assessed by study authors)
- Ethnic group (e.g., Indigenous peoples; will be determined post hoc, depending on populations encountered in studies)
- Geographical location (e.g., rural vs. urban settings, by country/region)
- Age groups
- Gender/sex (according to how reported by study authors)
- Chronic illness
- Immigrant status of child or parent (as reported by study authors)
- Risk factors for depression (to be determined post-hoc, depending on combination of risk factors as reported in studies)
- Type of setting

#### Sensitivity analyses

When appropriate, a sensitivity analysis will be conducted by restricting analyses to studies assessed as low overall ROB or good quality. Additional analyses may also be performed to explore statistical heterogeneity, address decisions made regarding the handling of data (e.g., multiple observations in a small percentage of patients), the timing of publication, study design issues, or data requiring computation.

#### Small-study effects

For outcomes reported in 10 or more studies in a meta-analysis, publication bias will be assessed using a combination of graphical aids (e.g., funnel plots) and/or statistical tests (e.g., Egger regression test, Hedges-Olkin) [77, 80].

#### Grading the certainty of evidence and interpretation

We will assess the certainty of evidence using the GRADE approach. We will perform separate GRADE assessments for RCTs and non-randomized controlled studies. GRADE evidence profiles and summary of findings tables will be prepared for each of the critical and important outcomes using the GRADE framework to assess each domain (i.e., study limitations, imprecision, inconsistency, indirectness, and other considerations (including publication bias)) [52, 58].

The ERSC will undertake initial assessments of the GRADE domains, except for imprecision, and format the evidence according to GRADE guidance. Members of the guideline working group will have the opportunity to review those draft GRADE tables to determine imprecision thresholds for interpreting the importance of absolute effect sizes observed for outcomes. The ERSC plans to finalize the GRADE assessments incorporating those thresholds. Given the evolution of GRADE guidance at the time of the writing of this protocol, the formulation of GRADE narrative statements to represent the quantity, magnitude, and certainty of the evidence will be informed by available guidance [71, 81, 82].

The GRADE assessments will be piloted among reviewers and a senior methodologist for a sample of three to six outcomes that, if possible, will demonstrate diversity among data/information sets. Following the pilot phase, two reviewers will independently and together assess the certainty of the evidence in GRADEpro GDT, compare GRADE table outputs, and resolve disagreements by discussion or consulting a senior team member.

#### Software

We will use Cochrane Review Manager 5.3 (RevMan) to calculate effect estimates and conduct meta-analyses, where needed [83]. For all analyses not possible in RevMan, we will use Comprehensive Meta-Analysis [84]. GRADE assessments (including piloting) and corresponding tables will be produced in GRADEpro GDT [85].

## Discussion

The results of this review and the manuscript will be published in the “Canadian Task Force on Preventive Health Care Evidence Reviews” thematic series. Our systematic review will provide an evidence base for the Task Force and others to develop guidance on screening for depression in children and adolescents in primary care settings in Canada. We will address considerations for future research and highlight the implications for primary care practice.

## Supplementary Information

**Additional file 1:.** DSM-5 and ICD-10 definition of MDE.docx (19 KB)

**Additional file 2:.** PRISMA-P 2015 checklist.docx (21 KB)

**Additional file 3:.** Search strategies.docx (27 KB)

**Additional file 4:.** Draft screening forms.docx (19 KB)

**Additional file 5:.** Draft data extraction form.docx (16 KB)

**Additional file 6:.** Stakeholder and peer review feedback.docx (24 KB)

## Data Availability

Not applicable.

## Abbreviations

DSM-5: Diagnostic and statistical manual of mental disorders, 5^th^ edition
ERSC: Evidence review and synthesis centre
GRADE: Grading of recommendations assessment, development and evaluation
ICD-10: International classification of diseases, 10^th^ edition
KQ: Key question
MDE: Major depressive episode
MDD: Major depressive disorder
NOS: Newcastle-Ottawa scale
PROSPERO: International prospective registry of systematic reviews
RCT: Randomized controlled trial
ROB: Risk of bias
USPSTF: United States Preventive Services Task Force
