# Supplementary material for: Screening for depression in children and adolescents: a protocol for a systematic review update
Source: Syst Rev. 2021 Jan 12;10:24. doi: 10.1186/s13643-020-01568-3 (PMC7802305; doi:10.1186/s13643-020-01568-3)
Supplement: Supplementary file 1 — Additional file 1:. DSM-5 and ICD-10 definition of MDE.docx (19 KB) [file 13643_2020_1568_MOESM1_ESM.docx]

## Additional file 1: DSM-5 and ICD-10 definition of major depressive episode.

| **DSM-5^a^ - Major Depressive Episode** | **ICD-10^b^ - Depressive Episode** |
| --- | --- |
| **Criteria that must be met** | |
| [A] Five or more of the symptoms listed below (i-ix) have been present during the same two-week period and represent a change from previous functioning. At least one of the symptoms is either (i) depressed mood or (ii) loss of interest or pleasure.  [B] The symptoms cause clinically significant distress or impairment in social, occupational, or other important areas of functioning;  [C] The episode is not attributable to the physiological effects of a substance or another medical condition.  **Criteria A-C represent a major depressive episode**  [D] The occurrence of the major depressive episode is not better explained by schizoaffective disorder, schizophrenia, schizophreniform disorder, delusional disorder, or other specified and unspecified schizophrenia spectrum and other psychotic disorders;  [E] There has never been a manic episode or a hypomanic episode.  **Criteria A-E define major depressive disorder** | [1] The duration of the whole episode should usually last at least two weeks, but if the symptoms are particularly severe and of very rapid onset, it may be justified to make this diagnosis after less than two weeks.  At least two of the following should be present:  [a] depressed mood  [b] loss of interest and enjoyment  [c] reduced energy leading to increased fatigability and diminished activity |
| **Additional criteria to be met** | |
| [i] Depressed mood most of the day, nearly every day, as indicated by either subjective report (e.g., feels sad, empty, hopeless) or observation made by others (e.g., appears tearful). (Note: **In children and adolescents, can be irritable mood)**  [ii] Markedly diminished interest or pleasure in all, or almost all, activities most of the day, nearly every day (as indicated by either subjective account or observation). | *[a] and [b] above in criteria that must be met.*  Additionally, at least two to four [i, ii, iii, iv, v, vi, vii] must be met |
| [iii] Significant weight loss when not dieting or weight gain (e.g., a change of more than 5% of body weight in a month), or decrease or increase in appetite nearly every day. (Note: **In children, consider failure to achieve expected weight gain)** | [i] Diminished appetite |
| [iv] Insomnia or hypersomnia nearly every day. | [ii] Disturbed sleep |
| [v] Psychomotor agitation or retardation nearly every day (observable by others, not merely subjective feelings of restlessness or being slowed down). | *[c] above in criteria that must be met* |
| [vi] Fatigue or loss of energy nearly every day. | *[c] above in criteria that must be met* |
| [vii] Feelings of worthlessness or excessive or inappropriate guilt (which may be delusional) nearly every day (not merely self-reproach or guilt about being sick). | [iii] Ideas of guilt and unworthiness |
| [viii] Diminished ability to think or concentrate, or indecisiveness, nearly every day (either by subjective account or as observed by others). (Note: **In children, a precipitous drop in grades may reflect poor concentration)** | [iv] Reduced concentration and attention |
| [ix] Recurrent thoughts of death (not just fear of dying), recurrent suicidal ideation without a specific plan, or a suicide attempt or a specific plan for committing suicide. | [v] Ideas or acts or self-harm or suicide |
|  | [vi] Bleak and pessimistic views of the future |
|  | [vii] Reduced self-esteem and self-confidence |

^a^ *Diagnostic and Statistical Manual of Mental Disorders, Fifth Edition*

^b^ “Depressive Episodes” in the *ICD-10 Classification of Mental and Behavioural Disorders. Clinical descriptions and diagnostic guidelines*
