# Supplementary material for: Screening for depression in children and adolescents: a protocol for a systematic review update
Source: Syst Rev. 2021 Jan 12;10:24. doi: 10.1186/s13643-020-01568-3 (PMC7802305; doi:10.1186/s13643-020-01568-3)
Supplement: Supplementary file 3 — Additional file 3:. Search strategies.docx (27 KB) [file 13643_2020_1568_MOESM3_ESM.docx]

## Additional file 3: Search strategies.

### Multi-database search strategy (RCTs)

Databases: Embase Classic+Embase, Ovid MEDLINE(R) ALL, PsycINFO, EBM Reviews - Cochrane Central Register of Controlled Trials

Search Strategy:

--------------------------------------------------------------------------------

1 exp Depressive Disorder/

2 Depression/

3 depress*.tw,kf.

4 dysthym*.tw,kf.

5 blues.tw,kf.

6 melanchol*.tw,kf.

7 MDD.tw,kf.

8 or/1-7 [GENERAL DEPRESSION]

9 Mass Screening/

10 (screen* or detect*).tw,kf.

11 (identif* or recogni*).ti.

12 ((early or earlier or earliest) adj5 (identif* or recogni*)).tw,kf.

13 (case finding? or casefinding?).tw,kf.

14 or/9-13 [GENERAL SCREENING]

15 8 and 14

16 Adolescent/

17 Child/

18 Minors/

19 (boy or boys or girl or girls or child* or school-age* or adolescen* or teen or teens or teenage* or youth or youths or highschool* or high-school* or juvenil* or pubescen* or under?age*).tw,kf.

20 ((age? or years) adj1 ("18" or eighteen)).tw,kf.

21 p?ediatric*.tw,kf.

22 or/16-21 [CHILD/ADOLESCENT POPULATION]

23 15 and 22 [SCREENING - DEPRESSION - CHILD/ADOLESCENTS]

24 exp Animals/ not Humans/

25 23 not 24 [ANIMAL-ONLY REMOVED]

26 (comment or editorial or news or newspaper article).pt.

27 (letter not (randomized controlled trial and letter)).pt.

28 25 not (26 or 27) [OPINION PIECES REMOVED]

29 (controlled clinical trial or randomized controlled trial or pragmatic clinical trial).pt.

30 clinical trials as topic.sh.

31 exp Randomized Controlled Trials as Topic/

32 (randomi#ation? or randomi#ed or randomly or RCT$1 or placebo*).tw,kf.

33 ((singl* or doubl* or trebl* or tripl*) adj (mask* or blind* or dumm*)).tw,kf.

34 trial.ti.

35 or/29-34

36 28 and 35 [RCTs]

37 (2017* or 2018* or 2019*).dt.

38 36 and 37 [UPDATE PERIOD]

39 38 use medall [MEDLINE RECORDS]

40 exp Depressive Disorder/

41 depress*.tw,kw.

42 dysthym*.tw,kw.

43 blues.tw,kw.

44 melanchol*.tw,kw.

45 MDD.tw,kw.

46 or/40-45 [GENERAL DEPRESSION]

47 screening/

48 mass screening/

49 screening test/

50 (screen* or detect*).tw,kw.

51 (identif* or recogni*).ti.

52 ((early or earlier or earliest) adj5 (identif* or recogni*)).tw,kw.

53 (case finding? or casefinding?).tw,kw.

54 or/47-53

55 depression assessment/

56 46 and 54

57 55 or 56 [DEPRESSION & SCREENING/ASSESSMENT]

58 adolescent/

59 child/

60 school child/

61 "minor (person)"/

62 (boy or boys or girl or girls or child* or school-age* or adolescen* or teen or teens or teenage* or youth or youths or highschool* or high-school* or juvenil* or pubescen* or under?age*).tw,kw.

63 ((age? or years) adj1 ("18" or eighteen)).tw,kw.

64 p?ediatric*.tw,kw.

65 or/58-64 [CHILD/ADOLESCENT POPULATION]

66 57 and 65 [DEPRESSION - SCREENING - CHILD/ADOLESCENT POPULATION]

67 exp animal/ or exp animal experimentation/ or exp animal model/ or exp animal experiment/ or nonhuman/ or exp vertebrate/

68 exp human/ or exp human experimentation/ or exp human experiment/

69 67 not 68

70 66 not 69 [ANIMAL-ONLY REMOVED]

71 editorial.pt.

72 letter.pt. not (randomized controlled trial/ and letter.pt.)

73 70 not (71 or 72) [OPINION PIECES REMOVED]

74 randomized controlled trial/ or controlled clinical trial/

75 exp "clinical trial (topic)"/

76 (randomi#ation? or randomi#ed or randomly or RCT$1 or placebo*).tw,kw.

77 ((singl* or doubl* or trebl* or tripl*) adj (mask* or blind* or dumm*)).tw,kw.

78 trial.ti.

79 or/74-78

80 73 and 79 [RCTs]

81 (2017* or 2018* or 2018*).dc.

82 80 and 81 [UPDATE PERIOD]

83 82 use emczd [EMBASE RECORDS]

84 exp Depressive Disorder/

85 Depression/

86 depress*.ti,ab,kw.

87 dysthym*.ti,ab,kw.

88 blues.ti,ab,kw.

89 melanchol*.ti,ab,kw.

90 MDD.ti,ab,kw.

91 or/84-90 [GENERAL DEPRESSION]

92 Mass Screening/

93 (screen* or detect*).ti,ab,kw.

94 (identif* or recogni*).ti.

95 ((early or earlier or earliest) adj5 (identif* or recogni*)).ti,ab,kw.

96 (case finding? or casefinding?).ti,ab,kw.

97 or/92-96 [GENERAL SCREENING]

98 91 and 97

99 Adolescent/

100 Child/

101 Minors/

102 (boy or boys or girl or girls or child* or school-age* or adolescen* or teen or teens or teenage* or youth or youths or highschool* or high-school* or juvenil* or pubescen* or under?age*).ti,ab,kw.

103 ((age? or years) adj1 ("18" or eighteen)).ti,ab,kw.

104 p?ediatric*.ti,ab,kw.

105 or/99-104 [CHILD/ADOLESCENT POPULATION]

106 98 and 105 [SCREENING - DEPRESSION - CHILD/ADOLESCENTS]

107 (2017* or 2018* or 2019*).up.

108 106 and 107 [UPDATE PERIOD]

109 108 use cctr [COCHRANE RECORDS]

110 "Depression (Emotion)"/

111 exp Major Depression/

112 depress*.tw.

113 dysthym*.tw.

114 blues.tw.

115 melanchol*.tw.

116 MDD.tw.

117 or/110-116 [GENERAL DEPRESSION]

118 Screening/

119 exp Screening Tests/

120 exp Health Screening/

121 (screen* or detect*).tw.

122 (identif* or recogni*).ti.

123 ((early or earlier or earliest) adj5 (identif* or recogni*)).tw.

124 (case finding? or casefinding?).tw.

125 or/118-124 [GENERAL SCREENING]

126 117 and 125 [DEPRESSION - SCREENING]

127 (boy or boys or girl or girls or child* or school-age* or adolescen* or teen or teens or teenage* or youth or youths or highschool* or high-school* or juvenil* or pubescen* or under?age*).tw.

128 ((age? or years) adj1 ("18" or eighteen)).tw.

129 p?ediatric*.tw.

130 or/127-129

131 126 and 130

132 limit 126 to (180 school age <age 6 to 12 yrs> or 200 adolescence <age 13 to 17 yrs>) [Limit not valid in Embase,Ovid MEDLINE(R),Ovid MEDLINE(R) Daily Update,Ovid MEDLINE(R) In-Process,Ovid MEDLINE(R) Publisher,CCTR; records were retained]

133 131 or 132 [DEPRESSION - SCREENING -CHILD/ADOLESCENT POPULATION]

134 Clinical Trials/

135 (randomi#ation? or randomi#ed or randomly or RCT$1 or placebo*).tw.

136 ((singl* or doubl* or trebl* or tripl*) adj (mask* or blind* or dumm*)).tw.

137 trial.ti.

138 or/134-137

139 133 and 138 [RCTs]

140 (2017* or 2018* or 2019*).up.

141 139 and 140 [UPDATE PERIOD]

142 141 use medall,emczd,cctr

143 141 not 142 [PSYCINFO RECORDS]

144 39 or 83 or 109 or 143 [ALL DATABASES]

145 remove duplicates from 144 [TOTAL UNIQUE RECORDS]

146 145 use medall [MEDLINE UNIQUE RECORDS]

147 145 use emczd [EMBASE UNIQUE RECORDS]

148 145 use cctr [CENTRAL UNIQUE RECORDS]

149 145 not (146 or 147 or 148) [PSYCINFO UNIQUE RECORDS]

***************************

CINAHL

| # | Query |
| --- | --- |
| S31 | S29 AND S30 |
| S30 | EM 2017-2019 |
| S29 | S21 AND S28 |
| S28 | S22 OR S23 OR S24 OR S25 OR S26 OR S27 |
| S27 | TI ( ((age or aged or ages or years) N1 ("18" or eighteen)) ) OR AB ( ((age or aged or ages or years) N1 ("18" or eighteen)) ) |
| S26 | TI ( pediatric* or paediatric* ) OR AB ( pediatric* or paediatric* ) |
| S25 | TI ( boy or boys or girl or girls or child* or (school w0 age*) or adolescen* or teen or teens or teenage* or (teen W0 age*) or youth or youths or highschool* or (high W0 school*) or juvenil* or pubescen* or underage* or (under W0 age*) ) OR AB ( boy or boys or girl or girls or child* or (school w0 age*) or adolescen* or teen or teens or teenage* or (teen W0 age*) or youth or youths or highschool* or (high W0 school*) or juvenil* or pubescen* or underage* or (under W0 age*) ) |
| S24 | (MH "Minors (Legal)") |
| S23 | (MH "Child") |
| S22 | (MH "Adolescence+") |
| S21 | S14 AND S20 |
| S20 | S15 OR S16 OR S17 OR S18 OR S19 |
| S19 | TI trial |
| S18 | TI ( ((singl* or doubl* or trebl* or tripl*) N1 (mask* or blind* or dumm*)) ) OR AB ( ((singl* or doubl* or trebl* or tripl*) N1 (mask* or blind* or dumm*)) ) |
| S17 | TI ( randomi?ed or randomly or RCT or RCTs or placebo* ) OR AB ( randomi?ed or randomly or RCT or RCTs or placebo* ) |
| S16 | (MH "Double-Blind Studies") OR (MH "Single-Blind Studies") OR (MH "Triple-Blind Studies") |
| S15 | (MH "Randomized Controlled Trials") |
| S14 | S7 AND S13 |
| S13 | S8 OR S9 OR S10 OR S11 OR S12 |
| S12 | TI ( "case finding" or "case findings" or casefinding or casefindings ) OR AB ( "case finding" or "case findings" or casefinding or casefindings ) |
| S11 | TI ( ((early or earlier or earliest) N5 (identif* or recogni*)) ) OR AB ( ((early or earlier or earliest) N5 (identif* or recogni*)) ) |
| S10 | TI (identif* or recogni*) |
| S9 | TI ( (screen* or detect*) ) OR AB ( (screen* or detect*) ) |
| S8 | (MH "Health Screening") |
| S7 | S1 OR S2 OR S3 OR S4 OR S5 OR S6 |
| S6 | TI MDD OR AB MDD |
| S5 | TI melanchol* OR AB melanchol* |
| S4 | TI blues OR AB blues |
| S3 | TI dysthym* OR AB dysthym* |
| S2 | TI depress* OR AB depress* |
| S1 | (MH "Depression+") |

### Multi-database search strategy (Controlled studies without random assignment)

Databases: Embase Classic+Embase, Ovid MEDLINE(R) ALL, PsycINFO

Search Strategy:

--------------------------------------------------------------------------------

1 exp Depressive Disorder/ (576156)

2 Depression/ (501370)

3 depress*.tw,kf. (1379963)

4 dysthym*.tw,kf. (11186)

5 blues.tw,kf. (4755)

6 melanchol*.tw,kf. (12067)

7 MDD.tw,kf. (41316)

8 or/1-7 [GENERAL DEPRESSION] (1590381)

9 Mass Screening/ (159734)

10 (screen* or detect*).tw,kf. (6781102)

11 (identif* or recogni*).ti. (876388)

12 ((early or earlier or earliest) adj5 (identif* or recogni*)).tw,kf. (183645)

13 (case finding? or casefinding?).tw,kf. (13324)

14 or/9-13 [GENERAL SCREENING] (7623016)

15 8 and 14 (161908)

16 Adolescent/ (3605434)

17 Child/ (3577861)

18 Minors/ (3154)

19 (boy or boys or girl or girls or child* or school-age* or adolescen* or teen or teens or teenage* or youth or youths or highschool* or high-school* or juvenil* or pubescen* or under?age*).tw,kf. (5064557)

20 p?ediatric*.tw,kf. (923183)

21 or/16-20 [CHILD/ADOLESCENT POPULATION] (8257782)

22 15 and 21 [SCREENING - DEPRESSION - CHILD/ADOLESCENTS] (32518)

23 exp Animals/ not Humans/ (18132939)

24 22 not 23 [ANIMAL-ONLY REMOVED] (24708)

25 (comment or editorial or news or newspaper article).pt. (2018679)

26 (letter not (randomized controlled trial and letter)).pt. (2155273)

27 24 not (25 or 26) [OPINION PIECES REMOVED] (24601)

28 controlled clinical trial.pt. (93519)

29 Controlled Clinical Trials as Topic/ (14679)

30 (control* adj2 trial).tw,kf. (380916)

31 Non-Randomized Controlled Trials as Topic/ (11105)

32 (nonrandom* or non-random* or quasi-random* or quasi-experiment*).tw,kf. (139717)

33 (nRCT or nonRCT or non-RCT).tw,kf. (796)

34 Controlled Before-After Studies/ (217085)

35 (control* adj3 ("before and after" or "before after")).tw,kf. (10653)

36 Control Groups/ (125694)

37 (control* adj2 group?).tw,kf. (1287996)

38 trial.ti. (537452)

39 Comparative Study.pt. (1851402)

40 ((comparative or comparison) adj (study or studies)).tw,kf. (270904)

41 or/28-40 [STUDY DESIGN FILTER] (4236078)

42 27 and 41 (2874)

43 (2015* or 2016* or 2017* or 2018* or 2019*).dt. (6054666)

44 (2015* or 2016* or 2017* or 2018* or 2019*).dp. (7873507)

45 42 and 43 (470)

46 42 and 44 (674)

47 45 or 46 [DATE LIMITS APPLIED] (678)

48 47 use medall [MEDLINE RECORDS] (490)

49 exp Depressive Disorder/ (576156)

50 depress*.tw,kw. (1388316)

51 dysthym*.tw,kw. (11290)

52 blues.tw,kw. (4775)

53 melanchol*.tw,kw. (12159)

54 MDD.tw,kw. (41470)

55 or/49-54 [GENERAL DEPRESSION] (1573066)

56 screening/ (294412)

57 mass screening/ (159734)

58 screening test/ (75813)

59 (screen* or detect*).tw,kw. (6791906)

60 (identif* or recogni*).ti. (876388)

61 ((early or earlier or earliest) adj5 (identif* or recogni*)).tw,kw. (183767)

62 (case finding? or casefinding?).tw,kw. (13459)

63 or/56-62 (7663086)

64 depression assessment/ (1718)

65 55 and 63 (163181)

66 64 or 65 [DEPRESSION & SCREENING/ASSESSMENT] (164530)

67 adolescent/ (3605434)

68 child/ (3577861)

69 school child/ (357871)

70 "minor (person)"/ (602)

71 (boy or boys or girl or girls or child* or school-age* or adolescen* or teen or teens or teenage* or youth or youths or highschool* or high-school* or juvenil* or pubescen* or under?age*).tw,kw. (5067153)

72 p?ediatric*.tw,kw. (948097)

73 or/67-72 [CHILD/ADOLESCENT POPULATION] (8292001)

74 66 and 73 [DEPRESSION - SCREENING - CHILD/ADOLESCENT POPULATION] (33028)

75 exp animal/ or exp animal experimentation/ or exp animal model/ or exp animal experiment/ or nonhuman/ or exp vertebrate/ (52186488)

76 exp human/ or exp human experimentation/ or exp human experiment/ (40023229)

77 75 not 76 (12164957)

78 74 not 77 [ANIMAL-ONLY REMOVED] (32581)

79 editorial.pt. (1153940)

80 letter.pt. not (randomized controlled trial/ and letter.pt.) (2150111)

81 78 not (79 or 80) [OPINION PIECES REMOVED] (32437)

82 controlled clinical trial/ (557137)

83 "controlled clinical trial (topic)"/ (10496)

84 (control* adj2 trial).tw,kw. (384975)

85 (nonrandom* or non-random* or quasi-random* or quasi-experiment*).tw,kw. (139966)

86 (nRCT or nonRCT or non-RCT).tw,kw. (797)

87 (control* adj3 ("before and after" or "before after")).tw,kw. (10658)

88 control group/ (125694)

89 (control* adj2 group?).tw,kw. (1287754)

90 trial.ti. (537452)

91 comparative study/ (2723480)

92 ((comparative or comparison) adj (study or studies)).tw,kw. (269305)

93 or/82-92 [STUDY DESIGN FILTER] (5088721)

94 81 and 93 (4014)

95 (2015* or 2016* or 2017* or 2018* or 2019*).dc. (8927750)

96 (2015* or 2016* or 2017* or 2018* or 2019*).dp. (7873507)

97 94 and 95 (799)

98 94 and 96 (741)

99 97 or 98 [DATE LIMITS APPLIED] (1440)

100 99 use emczd [EMBASE RECORDS] (800)

101 "Depression (Emotion)"/ (139634)

102 exp Major Depression/ (189258)

103 depress*.tw. (1374315)

104 dysthym*.tw. (11156)

105 blues.tw. (4746)

106 melanchol*.tw. (12024)

107 MDD.tw. (41200)

108 or/101-107 [GENERAL DEPRESSION] (1422496)

109 Screening/ (294412)

110 exp Screening Tests/ (29626)

111 exp Health Screening/ (252947)

112 (screen* or detect*).tw. (6773232)

113 (identif* or recogni*).ti. (876388)

114 ((early or earlier or earliest) adj5 (identif* or recogni*)).tw. (183552)

115 (case finding? or casefinding?).tw. (13258)

116 or/109-115 [GENERAL SCREENING] (7702000)

117 108 and 116 [DEPRESSION - SCREENING] (152601)

118 (boy or boys or girl or girls or child* or school-age* or adolescen* or teen or teens or teenage* or youth or youths or highschool* or high-school* or juvenil* or pubescen* or under?age*).tw. (5013517)

119 p?ediatric*.tw. (908792)

120 118 or 119 (5358204)

121 117 and 120 (23854)

122 limit 117 to (180 school age <age 6 to 12 yrs> or 200 adolescence <age 13 to 17 yrs>) [Limit not valid in Embase,Ovid MEDLINE(R),Ovid MEDLINE(R) Daily Update,Ovid MEDLINE(R) In-Process,Ovid MEDLINE(R) Publisher; records were retained] (130133)

123 121 or 122 [DEPRESSION - SCREENING -CHILD/ADOLESCENT POPULATION] (132887)

124 (control* adj2 trial).tw. (377590)

125 (nonrandom* or non-random* or quasi-random* or quasi-experiment*).tw. (139545)

126 (nRCT or nonRCT or non-RCT).tw. (795)

127 (control* adj3 ("before and after" or "before after")).tw. (10646)

128 (control* adj2 group?).tw. (1287635)

129 Experiment Controls/ (913)

130 trial.ti. (537452)

131 ((comparative or comparison) adj (study or studies)).tw. (266093)

132 or/124-131 [STUDY DESIGN FILTER] (2256812)

133 123 and 132 (11097)

134 (2015* or 2016* or 2017* or 2018* or 2019*).up. (66806709)

135 (2015* or 2016* or 2017* or 2018* or 2019*).dp. (7873507)

136 133 and 134 (9895)

137 133 and 135 (2249)

138 136 or 137 [DATE LIMITS APPLIED] (9953)

139 138 use medall,emczd (9778)

140 138 not 139 [PSYCINFO RECORDS] (175)

141 48 or 100 or 140 [ALL DATABASES] (1465)

142 remove duplicates from 141 [TOTAL UNIQUE RECORDS] (1040)

143 142 use medall [MEDLINE UNIQUE RECORDS] (488)

144 142 use emczd [EMBASE UNIQUE RECORDS] (500)

145 142 not (143 or 144) [PSYCINFO UNIQUE RECORDS] (52)

***************************
