# Supplementary material for: Screening for depression in children and adolescents: a protocol for a systematic review update
Source: Syst Rev. 2021 Jan 12;10:24. doi: 10.1186/s13643-020-01568-3 (PMC7802305; doi:10.1186/s13643-020-01568-3)
Supplement: Supplementary file 4 — Additional file 4:. Draft screening forms.docx (19 KB) [file 13643_2020_1568_MOESM4_ESM.docx]

## Additional file 4: Draft screening forms.

### For RCTs

**Title and abstract screening**

1. Does this reference discuss depression screening in children (6 to 11 years) and adolescents (12 to 17 years)?

**🔿 Yes/unclear***

🔿 No

*Those answered yes/unclear will be passed through to full-text screening.

**Full-text screening**

1. Language of publication

**🔿 English or French**

🔿 Other _____________________

1. Is this article a randomized controlled trial/cluster randomized controlled trial?

**🔿 Yes**

🔿 **Relevant systematic review**

🔿 No (select one of the options: Narrative review, non-RCT, controlled before-after, interrupted times series, cohort, case-control, cross-sectional, case series, case report, or other publication type (editorial, commentary, note, letter, opinion)

🔿 Abstract

1. Does the population include children (6-11 years) and/or adolescents (12 to 17 years)?

**🔿 Yes**

🔿 No

**🔿 Mixed population (children/adolescents and general population)**

**🔿 Unclear (contact authors)**

1. [If question of 3 is mixed population] If this article includes a mixed population, do they provide separate children and/or adolescents outcome data?

**🔿 Yes**

🔿 No

1. Does this study determine patient eligibility and randomize patients prior to administering the screening test?

**🔿 Yes**

🔿 No

**🔿 Unclear (contact authors)**

1. Does the study provide similar depression management and treatment resources to patients who were identified as depressed via screening in the screening arm of the trial and patients in either the screening or non-screening arms of the trial who were identified as depressed via other methods (e.g., unaided clinician diagnosis, patient report)?

**🔿 Yes**

🔿 No

**🔿 Unclear (contact authors)**

Typically, these questions are nested. If an answer allows us to proceed in the inclusion criteria, the next question will appear. Those bolded would be those that would pass through to the following question.

### For controlled studies without random assignment (if required)

**Title and abstract screening**

1. Does this reference discuss depression screening in children (6 to 11 years) and adolescents (12 to 17 years)?

**🔿 Yes/unclear***

🔿 No

*Those answered yes/unclear will be passed through to full-text screening.

**Full-text screening**

1. Language of publication

**🔿 English or French**

🔿 Other _____________________

1. Is this article a controlled study without random assignment?

**🔿 Yes (comment on study design)**

🔿 **Relevant systematic review**

🔿 No (select one of the options: Interrupted times series, case-control, cross-sectional, case series, case report, narrative review, or other publication type (editorial, commentary, note, letter, opinion)

🔿 Abstract

1. Does the population include children (6-11 years) and/or adolescents (12 to 17 years)?

**🔿 Yes**

🔿 No

**🔿 Mixed population (children/adolescents and general population)**

**🔿 Unclear (contact authors)**

1. [If question of 3 is mixed population] If this article includes a mixed population, do they provide separate children and/or adolescents outcome data?

**🔿 Yes**

🔿 No

Typically, these questions are nested. If an answer allows us to proceed in the inclusion criteria, the next question will appear. Those bolded would be those that would pass through to the following question.
