# Supplementary material for: Screening for depression in children and adolescents: a protocol for a systematic review update
Source: Syst Rev. 2021 Jan 12;10:24. doi: 10.1186/s13643-020-01568-3 (PMC7802305; doi:10.1186/s13643-020-01568-3)
Supplement: Supplementary file 5 — Additional file 5:. Draft data extraction form.docx (16 KB) [file 13643_2020_1568_MOESM5_ESM.docx]

## Additional file 5: Draft data extraction form.

**Publication details:** Year of publication, language

**Characteristics of study**: Study design, country of study conduct, setting, sample size, number of centres [if applicable], duration of follow-up, study funding source

**Characteristics of population**: Age, sex, ethnicity, risk factors for depression, information regarding respondent bias/representativeness of the included population

**Details about the exposure/intervention**: Type of screening test performed, screening tool(s)/question(s) used, type of treatment provided

**Details about comparator**: Any additional services provided to comparator group, type of treatment provided

**Outcomes of interest**: Definitions, measurement methods, data, adjusted and unadjusted effect estimates, cluster correlation coefficients (where relevant), lost to follow-up

**Risk of bias / Newcastle-Ottawa**
