# Supplementary material for: Screening for depression in children and adolescents: a protocol for a systematic review update
Source: Syst Rev. 2021 Jan 12;10:24. doi: 10.1186/s13643-020-01568-3 (PMC7802305; doi:10.1186/s13643-020-01568-3)
Supplement: Supplementary file 6 — Additional file 6:. Stakeholder and peer review feedback.docx (24 KB) [file 13643_2020_1568_MOESM6_ESM.docx]

## Additional file 6: Stakeholder and peer review feedback.

1. **Stakeholder feedback and responses**

| **Question 1: Do the research questions address the clinically important issues?** | | |
| --- | --- | --- |
| **Replies** | **Reviewers Response** | **Authors Response** |
| Yes: 5  No: 1 | 1. No comments  2. The research questions are appropriate and clearly stated.  3. No comments  4. For the most part; however, the focus on how parents/guardians value outcomes that may occur from screening for depression in children and adolescents and the influence of these values on decisions about being screened only focuses on things like stigma or unnecessary anxiety as barriers to screening but there are also unique barriers to screening in Indigenous contexts, including fear/apprehension of mainstream health care services.  5. No comments  6. KQ1 allows consideration of opportunity costs of screening for MDD (given the lack of direct evidence of value) – ie. Consideration of harms due to reduced opportunity/capacity in primary care to screen for (or manage) other conditions that may have higher evidence-based return value. Rather than considering depression screening in isolation, this question allows for recognition that recommendations on child and adolescent depression screening occurs in a context of other health management considerations. A key consideration for integrated care to be achieved. KQ1a allows comparison of value for recommendations for general or higher-risk population screening, allowing for greater efficiency, reduced risk of inadvertent harm (alluded to in KQ2+2a) for populations where screening value may to be as reliably achieved. | 2. Thank you for your review.  4. We agree. We note that research in such populations often is carried out in a participatory action framework, and that the nature of healthcare provision may differ between people living on reserve and off reserve. If there has been an RCT or a non-randomized controlled study within a participatory action framework, this should now be identified in our literature search. Moreover, our protocol included consideration of subgroups by ethnicity, and indigenous people would be considered as a subgroup. We can make this clearer in the protocol by specifying Indigenous peoples as an example, and in the review itself, we will include consideration of indigenous and other vulnerable people in the Discussion.  6. Thank you for your review and this feedback. |
| **Question 2: Are there any important sources of evidence that we did not include that should be considered in our review? If yes, please provide the source.** | | |
| **Replies** | **Reviewers Response** | **Authors Response** |
| Yes: 0  No: 6 | 1. No comments  2. Very complete and appropriate sources of evidence.  3. No comments  4. None that can be specifically identified at the present time; however, the narrow scope of the review on RCTs may be biasing results to a particular cultural context to the exclusion of others.  5. No comments  6. No comments | 4. Thank you for this point. The Task Force had extensive discussion about whether or not to include non-randomized controlled studies in case we find no RCTs. We have agreed to add an additional search for non-randomized controlled studies. |
| **Question 3: Do you have any major concerns about the protocol that we should address?** | | |
| **Replies** | **Reviewers Response** | **Authors Response** |
| Yes: 3  No: 3 | 1. No comments  2. Very well researched and developed protocol, understanding that this is an update revie[w].  3. Line 509: Why P<0.1? Is this because it will include all P<0.05  Line 535: Which ethnicity will be used? First generation Canadians or second generation Canadians or anyone that has foreign roots?  Line 540: Based on my own recent studies, I found that disease prevalence differs depending on number of years after immigration or landing which I sub divided into <5 yrs, 5-10 and >10 years. Perhaps there could be a correlation also for these adolescent.  4. Given that Indigenous peoples are underrepresented in RCTs, the sole focus of the review on identifying RCTs related to research on depression screening among children and youth means that research on Indigenous children and youth will likely not be identified, leaving out a significant population among whom high rates of suicide and mental health issues have been identified. The protocol also emphasizes the focus on formal screening processes, without considering the appropriateness of both the content and methods of screening for culturally diverse contexts.  5. No comments  6. As noted above, may need to explicitly seek to capture concerns about opportunity costs/harm from general population MDD screening in children + adolescents, which is different than unintended direct harm from screening (which is what KQ2 alludes to). | 3. A cut-off of 0.10 rather than 0.05 is recommended practice in meta-analyses. In most cases, the statistical power of tests for heterogeneity are low because of the small sample size and/or few studies which may imply heterogeneity exists even if not statistically significant. I have added two references in the protocol.  We will use what the authors report ethnicity in their studies.  Thank you, this will be useful for the analysis. This will rely on what the study authors report, but we will consider this categorization if data is available.  4. Thank you for this feedback. We believe this has been addressed with the previous responses.  6. Thank you for your review. We will certainly look for opportunity costs. |

2. **Peer review feedback and responses**

| **Question 1: Do the research questions address the clinically important issues?** | | |
| --- | --- | --- |
| **Replies** | **Reviewers Response** | **Authors Response** |
| Yes: 4  No: 0 | 1. No comments  2. No comments  3. The research question seeks to focus on the identification of cases through screening that would not otherwise present or be identified. This is appropriate. The focus on benefits and harms is also appropriate as is the distinction between universal approaches and more targeted approaches (i.e. those identified as at risk in some way).  4. No comments | 3. Thank you. |
| **Question 2: Are there any important sources of evidence that we did not include that should be considered in our review? If yes, please provide the source.** | | |
| **Replies** | **Reviewers Response** | **Authors Response** |
| Yes: 1  No: 3 | 1. No comments  2. This review builds on the Roseman review of 2017 but now adds a new question; is screening effective for those at risk of depression. this question was not included in the Roseman review which went back to 2006. To really update the Roseman review it might be important to do a lit search on studies that address KQ1a back to 2006. Otherwise its not really a comprehensive addition to the Roseman review.  3. The approach to searching the literature covers the appropriate sources.  4. No comments | 2. Thank you for highlighting this issue. After discussion with authors (Dr. Brett Thombs is corresponding author), we confirm that this review did include a search for evidence from high-risk populations but did not directly specify this. If studies in high-risk populations had been identified, these would have been reported, but no evidence was identified. The working group concludes that a specific search relating to KQ1a back to 2006 is not needed.  3. Thank you. |
| **Question 3: Do you have any major concerns about the protocol that we should address?** | | |
| **Replies** | **Reviewers Response** | **Authors Response** |
| Yes: 1  No: 3 | 1. No comments  2. I think the introduction should include a more systematic approach to the review of depression guidelines that discuss screening. The authors mention the US task force, and GLAD-P but not NICE, AACAP Practice Parameters (which I believe have new guidelines), CANMAT, Beyond Blue etc. Also I was unclear as to what constitutes a non-mental health setting? Child welfare? Youth justice, schools? Indigenous settings? The list is potentially quite long. I know there are some studies of screening and treatment in schools that show evidence of efficacy. Are these relevant? I do want to say though that the methodology of the protocol is very strong and thorough. No concerns there.  3. I have no major concerns. The proposed review is methodologically robust. I have one suggestion about potential points to address. In the introduction it may be useful to make the case more explicitly for the decision to focus on RCT evidence of screening rather than indirect evidence as used in other guidance. At the moment, the rationale for this is left a little implicit.  4. Excellent protocol; very clear methods. | 1. Thank you for your review.  2. We limited to evidence-based guidelines, most of these other guidelines are not based on systematic review evidence or are focussed on adults. We will clarify this in the protocol.  We will more explicitly define what types of settings are of interest. We are interested in settings where screening is performed by a health professional outside of mental health, treatment facilities, and psychiatric settings.  3. Thank you for this point. The Task Force had extensive discussion about whether or not to include non-randomized controlled studies in case we find no RCTs. The working group has decided to add an additional search non-randomized controlled studies.  4. Thank you for your review. |
